# Changes in BMI-distribution from 1966–69 to 1995–97 in adolescents. The Young-HUNT study, Norway

**DOI:** 10.1186/1471-2458-7-279

**Published:** 2007-10-04

**Authors:** Sigrid Bjørnelv, Stian Lydersen, Arnstein Mykletun, Turid Lingaas Holmen

**Affiliations:** 1 HUNT Research Centre, Department of Public Health and General Practice, Faculty of Medicine, Norwegian University of Science and Technology, Neptunvn 1, 7650 Verdal, Norway; 2 Department of Psychiatry, Levanger Hospital, Nord-Trøndelag Hospital Trust, 7600 Levanger, Norway; 3 Unit for Applied Clinical Research, Department of Cancer research and Molecular Medicine, Norwegian University of Science and Technology, 7491 Trondheim, Norway; 4 Faculty of Psychology, University of Bergen, 5020 Bergen, Norway

## Abstract

**Background:**

The aim of this study was to explore changes in the BMI-distribution over time among Norwegian adolescents.

**Methods:**

Height and weight were measured in standardised ways and BMI computed in 6774 adolescents 14–18 years who participated in the Young-HUNT study, the youth part of the Health-study of Nord-Trondelag County, Norway in 1995–97. The results were compared to data from 8378 adolescents, in the same age group and living in the same geographical region, collected by the National Health Screening Service in 1966–69.

**Results:**

From 1966–69 to 1995–97 there was an increased dispersion and a two-sided change in the BMI-distribution. Mean BMI did not increase in girls aged 14–17, but increased significantly in 18 year old girls and in boys of all ages. In both sexes and all ages there was a significant increase in the upper percentiles, but also a trend towards a decrease in the lowest percentiles. Height and weight increased significantly in both sexes and all ages.

**Conclusion:**

The increased dispersion of the BMI-distribution with a substantial increase in upper BMI-percentiles followed the same pattern seen in other European countries and the United States. The lack of increase in mean BMI among girls, and the decrease in the lowest percentiles has not been acknowledged in previous studies, and may call for attention.

## Background

Height, weight and Body Mass Index (BMI) have been used extensively as indicators of weight-related health-problems. Changes in these anthropometric characteristics reflect different changes in society as well as in its individuals. Better nutrition increases both height and weight, and reduces health problems connected with malnutrition. Increased weight and BMI might also indicate a non-healthy diet and too little physical activity causing health problems, while low weight and BMI might reflect unhealthy dieting and eating problems. In order to prevent and modify any unwanted changes in weight and BMI, it is important to observe trends in the BMI-distribution over time, different changes may call for different or new prevention strategies [1].

Studies both from Europe [2-7] and the United States [8,9] have shown increasing BMI-values in adolescents during the last two decades, with a marked increase in the highest BMI-percentiles. However, some studies [10,11] do not support an overall increase in BMI-percentiles, indicating than not all adolescents belong to the population with increasing BMI. At the same time different studies reports high prevalence of dieting and other unhealthy weight control behaviours in this age group [12,13]. One recent study [3] have focused on the increased dispersion of the BMI-distribution, but surveying Medline 1994-May 2007 for the descriptors BMI and change limited to adolescents, we found no study focusing on changes in the lowest BMI-percentiles.

This article aimed to study changes over time in height, weight, BMI and the total BMI-distribution, by comparing measurements 30 years apart from same aged adolescents, living in the same region.

## Methods

### Study populations

Data are collected from two different study-populations from the county of Nord-Trondelag, Norway in 1966–69 and 1995–97. The county consists of rural and industrial areas with small social differences and sociodemographic changes between the two studies. In 1995 the county had 127,000 inhabitants, 10,000 more than in 1969.

#### 1) The National Health Screening Service (NHS) 1966–69

The Norwegian National Health Screening Service (NHS) was established in 1940, and from 1952 on regularly basis collected population data from the whole country [14]. As part of this program, standardised data on height and weight was collected from the population of Nord-Trøndelag County in 1966–69. Totally 80% (8378) of the adolescent population 14–18 years old participated, 4372 boys and 4006 girls.

#### 2) Young-HUNT, 1995–97

Young-HUNT is the youth part of Nord-Trondelag Health Study (HUNT)[15]. All students in Junior High Schools (13–16 years) and High Schools (16–19 years) in the county were invited to the Young-HUNT study. The response rate was 90%. A self reported questionnaire was completed during one school hour. In the clinical part of the study, specially trained nurses measured weight and height within one month after completion of the questionnaire. In the age group 14–18 years, height and weight were measured in 3307 boys and 3367 girls, totally 6674 adolescents.

### Measurements

#### Age

In both studies age is reported according to truncated age, that is, age 14 ranges from 14.00 – 14.99 years. In both populations and for all ages in both boys and girls, the mean age ranged from 0.47 to 0.52 years higher than the truncated age.

Mainly due to differences in birth rates, the number of participants in each age group differs in and between the two study populations. The number of participants is lower in the highest ages. In the Young- HUNT study students in High-school having a year of apprenticeship outside of school were not invited. In 1966/69, many of the oldest adolescents had ended school and worked outside the county.

#### Height and weight

The Young-HUNT study followed the procedures for measuring height and weight made by the NHS. Height and weight were measured by trained nurses using internally standardised meter measures and weight scales. The subjects wore light clothes (T-shirts and trousers) without shoes. Height was read to the nearest cm and weight to the nearest half kilo or kilo. In our calculations of measurement error, we have assumed that weight was rounded off to the nearest kg.

### Statistical analyses

The differences in mean height, weight and BMI were analysed using Student's t-test with unequal variances. Analyses of log transformed BMI values gave the same results (not reported here). P-values for differences in the BMI-percentiles between the studies were computed using bootstrapping with 1000 replications and the "bias corrected and accelerated" (Bca) method [16]. Bootstrapping was used because BMI was not normally distributed, especially in the tails of the distribution. Measurement error in change in BMI percentiles due to rounding off measurements to the nearest kg and cm was computed using Monte Carlo simulations with m = 1000 replications. The potential bias in BMI due to age truncation was calculated by linear interpolation. The statistical programs S-PLUS (Insightful Corporation) and R were used for bootstrapping and Monte Carlo Simulations. SPSS version 12.0 (SPSS Inc., Chicago) was used for the other analyses. Two sided p-values <0.05 were considered significant.

## Results

### Height and weight

Mean height and weight increased from 1966–69 to 1995–97. The differences were significant (p < 0.001) for all ages and both sexes (Tables 1 and 2).

**Table 1:** Distribution of height and weight for each sex and age group in the two study- populations NHS and Young-HUNT

**Age**	**N**		**Height**				**Weight**			
	**NHS (66/69)**	**Young- HUNT (95–97)**	**NHS (66/69)**		**Young- HUNT (95–97)**		**NHS (66/69)**		**Young- HUNT (95–97)**	

**Boys**			**Mean**	**SD**	**Mean**	**SD**	**Mean**	**SD**	**Mean**	**SD**

14	973	755	163.9	8.7	170.6	8.3	53.4	9.8	59.7	11.5
15	960	756	170.5	8.0	175.3	7.2	60.1	9.2	64.7	11.1
16	922	658	174.0	6.6	178.3	6.7	64.0	9.0	68.4	11.3
17	767	639	176.3	6.5	180.1	6.5	67.3	8.2	72.5	12.0
18	750	499	177.0	6.6	180.1	7.0	69.7	8.3	74.4	12.2
**Girls**										
14	941	773	161.6	5.7	164.6	6.2	53.9	7.6	56.4	9.7
15	932	755	163.4	5.7	166.2	5.9	56.4	8.2	58.9	9.0
16	825	667	164.2	5.6	166.4	5.9	58.4	8.3	60.4	9.5
17	691	610	164.7	5.7	166.7	6.2	59.7	7.9	62.0	9.7
18	617	542	164.9	5.5	167.3	5.7	60.4	8.1	63.7	10.8

**Table 2:** Distribution of BMI for each sex and age group in the two study- populations NHS and Young- HUNT

**Age**	**Mean Body Mass Index**					
	**NHS (66/69)**		**Young-HUNT (95/97)**		**Difference**	

	**Mean**	**SD**	**Mean**	**SD**	**Diff**.	**P**

**Boys**						
14	19.7	2.3	20.4	3.1	0.7	<.001
15	20.6	2.2	21.0	2.9	0.4	<.001
16	21.1	2.3	21.5	3.0	0.4	<.001
17	21.6	2.1	22.3	3.3	0.7	<.001
18	22.2	2.2	22.9	3.3	0.7	<.001
**Girls**						
14	20.6	2.4	20.8	3.1	0.2	.261
15	21.1	2.7	21.3	3.0	0.2	.147
16	21.6	2.7	21.8	3.2	0.2	.261
17	22.0	2.7	22.3	3.1	0.3	.061
18	22.2	2.6	22.8	3.6	0.6	.002

Height and weight increased more in boys compared to girls. For boys, height increased most in the youngest and mean height reached maximum in younger age in 1995/97 compared to 1966/69. No significant age dependence was found for height increase in girls or for weight-increase in either sex.

### Mean BMI

In girls 14–17 years there was no significant increase in mean BMI from 1966/69 to 1995/97, the increase was significant only in the 18 years old (p = 0.002). Among boys, mean BMI increased significantly (p < 0.001) in all ages (Tables 1 and 2).

### BMI distribution

**Figure 1:**
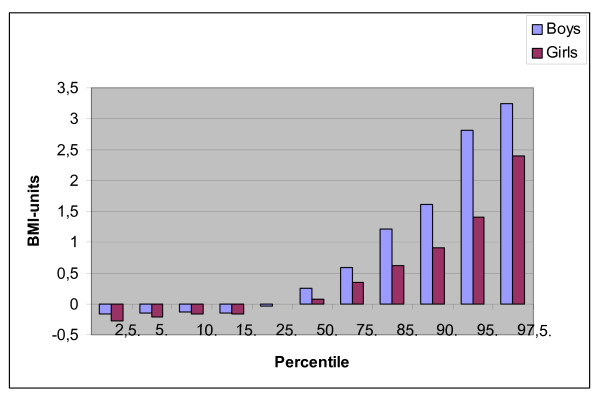
Changes in the BMI-distribution 1966/69 – 1995/97.

The change in the BMI-distribution showed an increased dispersion in BMI-values from 1966/69 to 1995/97 (Tables 1 and 2, Figure 1). There was a significant and skewed increase in the upper percentiles, with a shift around the 50^th^-percentile, but a trend for a decrease in the lower percentiles. Although significant only in girls aged 14 and boys aged 16 and 18, the pattern of decrease was consistent for all the lower percentiles in both sexes and all ages (Table 3). If there was no expected decrease in the lower percentiles, the probability of observing a decrease for all 10 age-sex groups would be (1/2)^10 ^= 0.001. Hence, the observed reduction in the lower percentiles of BMI as a whole is highly significant.

**Table 3:** Differences in values of BMI-percentiles (in BMI-units) between the two study populations NHS (1966/69) and Young-HUNT (1995/97)^1^

	Boys					Girls				
	
Age	14	15	16	17	18	14	15	16	17	18
Percentiles(a)										
2.5	-0.2	-0.16	-0.43*	-0.55	-0.87*	-0.6*	-0.28	-0.07	-0.04	-0.07
5	-0.11	-0.14	-0.18	-0.14	-0.93*	-0.45*	-0.36	-0.24	-0.12	0
10	0	-0.15	-0.09	-0.10	-0.59*	-0.39*	-0.03	-0.26	-0.09	0.02
15	-0.01	-0.16	-0.11	-0.04	-0.15	-0.37	-0.08	-0.32	0	0.09
25	0.08	-0.10	-0.17	0.15	0.11	-0.28	0.03	-0.1	0.14	0.04
50	0.28*	-0.05	0.21	0.39*	0.35	-0.2	0.26	0.02	-0.09	0.19
75	0.94	0.43	0.49	0.77	0.97	0.36	0.16	0.13	0.54	0.51
85	1.62	1.08	0.83	1.14	1.74	0.89	0.08	0.57	1.79	1.14
90	2.28*	1.89*	1.29*	1.72*	2.26*	0.9*	0.53	0.96	1.03	1.73*
95	2.94*	2.36*	3.04*	2.89*	2.73*	1.53*	1.15	1.4*	1.72*	2.71*
97.5	3.33*	2.78*	3.31*	4.43*	5.08*	2.24*	0.75	2.13*	2.29*	4.38*

* p < .05 for mean difference equal to 0.

^1 ^Since height and weight were rounded off, to the nearest cm and half kg, there is measurement error in the tabulated differences. Its standard deviation has been computed as 0.07, 0.03, and 0.12, BMI-units respectively, for the 2.5, 50, and 97.5 percentiles.

Changes in the upper percentiles (90^th^, 95^th ^and 97.5^th^) were significant for boys in all ages, but for girls there were no significant changes in the 90^th ^percentile for ages 15–17. In absolute values, the changes in the uppermost percentiles were greater in boys than in girls (Table 3).

## Discussion

Our study confirmed a substantial increase in the upper BMI-percentiles over time in the adolescent population. However, no significant increases in mean BMI were found for girls 14–17 years, and the study showed an increased dispersion of the BMI-distribution with a consistent pattern of a decrease in the lowest BMI percentiles, a trend for both sexes and all ages. Previous studies have focused primarily on changes in the upper percentiles, missing these changes in the lower percentiles indicating that the increase in BMI does not involve all adolescents.

### Strengths and limitations

The strength of our study is that both NHS and Young-HUNT sampled data from the same county, including more than 80% of the adolescent population, and with the same standardized procedures for measurements of height and weight. Mean ages in all age- and sex groups were similar. We have no data on secular changes in the study periods, but in both studies data were collected over a restricted time period (2–4 years), reducing the probability of major secular changes within each survey period. The population of Nord-Trondelag County has been stable with small sociodemographic and ethnic differences. The large number of participants and high participation rates support a high external validity. Also, Nord-Trondelag County has a sex and age distribution similar to Norway as a whole. The same is true for geography, industry, sources of income and economy.

When comparing results from two cohorts studied 30 years apart, some potential biases need to be addressed. Despite using the same protocols for measurements, differences in measurement-accuracy, time since meal and clothing including seasonal variations could influence the results. No systematic biases are likely in these aspects. We have no reasons to believe that the weight measurements were performed differently in the two studies in respect to weight of light clothes and time since meal. Both studies were conducted during the school-day (08.30 – 14.00), and school-year, with no measurements in June, July and August.

Another issue is the effect of rounding off height and weight to the nearest cm and kg. However, testing this, the resulting uncertainties in BMI percentile differences, given in the footnote for Table 3, is too small to explain the observed differences in BMI-values. The consistent pattern across age-and gender groups, together with the lack of increase of mean BMI in girls 14–17 year, also support the findings of a decrease in the lowest BMI-percentiles.

Potential bias due to age truncation is practically negligible, as stated in the footnote for Table 3.

Although a marked increase in the upper BMI-percentiles is well established, not all studies support the notion of an overall increase in BMI. This may support our findings of a decrease in the lowest BMI-percentiles and no increase in mean BMI in girls 14–17 years. While the prevalence of overweight and obesity in British children increased from 1984 – 1994 [17], a later short report found no significant changes in mean BMI 1986–1996 for girls aged 12–16 [10]. Also an Australian study among primary school children showed no increase in mean BMI from 1985–1997 among girls 12 years old and boys 7, 8 and 10 years [18]. Two Swedish studies have different conclusions. One article comparing the values of the 50-percentile between two cohort studies in the 1950s and in the 1970s summarised that the Swedish child population had remained at a very similar BMI-level since the Second World War [11], another study concluded that the median BMI was significantly higher in 2001 than in 1987 for boys aged 10, 13 and 16 years, and in girls 16 years old [2]. Also in this latest study, the greatest change in BMI was found in the highest percentiles.

Comparing different studies of changes in BMI- percentile is complicated due to use of different methodology, including measurements, sampling procedures, the decade the study started, and time span between measurements. The fact that other studies conducted within different time frames also reports some decrease in the lower percentiles, can indicate that this not only is a Norwegian phenomenon. Studies from both Europe [2,4-7] and the United States [8,9] have all focused on the changes in upper percentiles. Few studies have evaluated changes in the entire BMI-distribution. A Finnish [5] study in age groups 12, 14, 16 and 18 years found, according to the tables, little or no change in the lower (5^th ^and 15^th^) and middle (50^th^) percentile. A Swedish study [3] evaluating the differences in BMI-distribution in children 2–15 years of age born in 1973/75 and 1985/87 found an increase only above the 25^th ^percentile, and more pronounced in the upper parts of the BMI-distribution. Girls 13 and 14 years old had lower values of the 5th percentile in 1985/87 compared to 1973/75. Different other studies [2,7-9] have also presented tables with decrease in the lowest percentile in a few age- and sex groups, but without any comment on these findings. Although statistically significant only in boys 16 and18 years and girls 14 years, the consistency of the pattern in all ages and both sexes in our study, indicates a clear trend of decrease in the lower percentile that should not be ignored.

Existing Norwegian standards for height and weight [19] in children and adolescents were constructed from data sampled in Bergen from 1971–74. No recent official measures of height and weight have been published, making it difficult to predict when and how fast the changes have occurred. British studies in adolescents have shown little change in the prevalence of overweight and obesity between 1974–1984, but a significant increase between 1984 and 1994 [10,17]. A follow-up study of Young- HUNT in 2000–01 with 1613 girls and boys aged 17 and 18 years found no significant changes in height and weight from the Young- HUNT studies 1995–97 (data not shown) indicating that changes mainly occurred before 1995. This assumption is supported by the adult part of the HUNT-study, showing a marked increases in BMI among young adults between HUNT-I (1984–86) and HUNT-II (1995–97) [20].

Changes in the BMI-distribution over 30 years, as shown in this study from Nord- Trondelag Norway, may have multiple different explanations, both biological and socio-cultural, and their clinical relevance should be explored. Better socioeconomic conditions may have led to two different problems among adolescents, on one side obesity on the other side unhealthy weight control behaviour including eating problems. Especially in girls dieting and eating problems was reported quite frequently in the Young-HUNT population [12]. The fact that mean BMI in our study showed no increase in girls 14–17 years supports the need to follow the changes in BMI -distribution, not only the highest percentiles,

## Conclusion

The increased dispersion in the BMI distribution from 1966/69 to 1995/97 with an increase in the highest percentile confirms the increased prevalence of obesity in the adolescent population found in other studies. The lack of increase in mean BMI in girls 14–17 years and a decrease in the lowest percentiles also calls for attention. When studying changes in BMI distribution and national differences, it is necessary not only to compare the mean or median, but also, as this study show, to look at the whole BMI-distribution. Focus should be on etiological factors determining the individual BMI as well as the shape of the total BMI-distribution.

## Competing interests

The author(s) declare that they have no competing interests.

## Authors' contributions

TLH is the Principal Investigator of the Young- HUNT studies, and has contributed to the planning and reporting of this as well to writing the article.

SL contributed to statistical analysis and to writing the article

AM has contributed to the planning and reporting of the work and writing the article.

SB is the guarantor.

## Pre-publication history

The pre-publication history for this paper can be accessed here:


